# Health system-related barriers and facilitators to tuberculosis preventive treatment: a qualitative case study comparing implementation in the Republic of Moldova and Georgia

**DOI:** 10.1136/bmjresp-2024-002802

**Published:** 2025-09-03

**Authors:** Zaruhi Mkrtchyan, Valentina Vilc, Maka Danelia, Askar Yedilbayev, Anna M Foss, Patrick Nguipdop-Djomo, Andrei Dadu

**Affiliations:** 1 Public Health Policy, London School of Hygiene & Tropical Medicine, London, UK; 2 Institute of Phthisiopneumology "Chiril Draganiuc”, Chisinau, Moldova (the Republic of); 3 National Center for Disease Control and Public Health, Tbilisi, Georgia; 4 World Health Organization Regional Office for Europe, Copenhagen, Denmark; 5 Epidemiology and Population Health, London School of Hygiene & Tropical Medicine, London, UK

**Keywords:** Tuberculosis, Infection Control

## Abstract

**Introduction:**

Despite WHO’s recommendations and the 2023–2030 Tuberculosis (TB) action plan, uptake of TB preventive treatment (TPT) remains suboptimal. In this paper, we use two countries of the WHO Europe Region, the Republic of Moldova and Georgia, that are at different stages of implementation of TB prevention policies, as a case study to examine health system barriers and facilitators to TPT scale-up.

**Methods:**

In this case study, we used methods of qualitative research—interviews with three stakeholder groups: health service providers and National TB Programme staff; civil society organisations and international partners or donors. The data were collected via videoconference, transcribed, then coded and analysed using NVivo V.14. Thematic analysis was conducted.

**Results:**

Facilitators for TPT delivery in both settings include an established TB clinical network, well-functioning communication systems and an uninterrupted supply of TPT medicines.

In both settings, healthcare providers generally exhibit positive attitudes towards treating TB infection; however, some remain sceptical and cautious, particularly regarding prescribing TPT without confirmation of TB infection, a challenge compounded by limited access to testing for TB infection. Evidence of TB infection is also important for patients’ decisions on initiation and adherence to treatment. Other barriers to effective service delivery of TPT include shortages and high workload of primary healthcare personnel, ambiguity in the role of family doctors in the management of TPT and low prioritisation of TPT during regular monitoring visits.

**Conclusions:**

The case study identified similar challenges in the rollout of TPT across both settings, highlighting common barriers hindering effective implementation. For optimal TPT rollout, enhancing provider confidence, improving access to testing for TB infection and strengthening integration with primary healthcare with refined roles of family doctors are essential. Both settings would also benefit from improved monitoring and evaluation systems and prioritisation of TB prevention in monitoring.

**WHAT IS ALREADY KNOWN ON THIS TOPIC**

- Healthcare system barriers to tuberculosis (TB) preventive treatment (TPT) include limited resources, poor integration of TB services with primary healthcare, and in low-incidence settings, deprioritisation of TPT.

**WHAT THIS STUDY ADDS**

- Using two countries at contrasting stages of the implementation process as case studies, we identified and documented health system-related challenges for scale-up of TPT within the WHO European Region.

**HOW THIS STUDY MIGHT AFFECT RESEARCH, PRACTICE OR POLICY**

- This study identifies health system challenges to the rollout of TPT and provides recommendations to support its scale-up across the WHO European Region.
- It highlights the need for better integration with primary healthcare, clearer roles of family doctors, improved access to testing for TB infection, increased provider confidence and enhanced monitoring systems for TB prevention.
- The findings are relevant to other settings that have similar TB epidemiology and health system structures, offering valuable guidance for research, policy-making and TB programme management in comparable contexts.

## Introduction

 Despite advances in prevention and treatment, tuberculosis (TB) remains a threat to global health, and its impact is particularly high in resource-limited settings.^1 2^ Noting the improving epidemiological trends and overcoming the challenges to national health systems caused by the pandemic, the WHO’s European Region was well positioned to begin developing a strategy for TB elimination. In 2022, the 72nd session of the WHO Regional Committee for Europe adopted a new regional TB action plan for 2023–2030, with recommendations and priority actions to meet the goal of becoming TB-free by 2030.^3^ One of the priority actions under this strategy is to expand TB preventive treatment (TPT)—key healthcare measure to safely reduce the likelihood of progression from TB infection (TBI) to TB disease.^4^,^7^

WHO recommends TPT for people living with HIV (PLHIV), household contacts of people diagnosed with TB and clinical risk groups using available treatment regimens^8 9^ and individual approaches to maximise treatment initiation and completion.^9 10^

Globally, the coverage of TPT in 2023 reached 56% for PLHIV and 21% for household contacts of TB patients. In the WHO European Region, the TPT coverage is slightly higher: 57% for PLHIV and 37% for TB contacts.^11^ However, these estimates remain below the target of 90%, committed by the Member States at the United Nations’ High Level Meeting on Tuberculosis.^3 12^

The factors contributing to suboptimal provision of TPT vary. From a patient perspective, challenges related to acceptance of TPT include fear of side effects, treatment duration, access to services and poor doctor–patient relationships.^13^,^15^ From the healthcare system perspective, barriers to TPT scale-up include deviation from or non-adherence to guidelines, access to testing and diagnostics, low integration between TB and primary healthcare (PHC) services.^16^,^19^ In low-incidence settings, deprioritisation of TBI is another challenge.^18 20^ An important factor is patient–provider interaction and how healthcare providers influence the patient decision-making related to acceptance of therapy.^13 14 21^

Den Boon *et al* highlight nine essential (efficacy, duration, dosage, formulation, frequency, ways of administration, safety, prevention of drug resistance and priority groups) and four desirable (cost, safety in special risk groups, treatment adherence and need for drug susceptibility testing) pharmaceutical and clinical aspects for effective TPT.^22^ To roll out TPT, Satyanarayana *et al* emphasise scaling up systematic screening of high-risk groups.^23^

This paper presents results of a secondary analysis of data on TPT rollout from a case study examining health system barriers and facilitators to implementing TB policies in two countries of the WHO European Region.

## Methods

### Study design and setting

The case study was conducted using methods of qualitative research in two countries, selected based on their different stages in the rollout of TB prevention policies and their similarities in socioeconomic development status, stage of healthcare reforms, health systems, key health indicators and TB epidemiology. We employed a non-random pragmatic approach to select the Republic of Moldova, as a country with operational TB policies, and Georgia, which is at an earlier stage of implementation.

The Phthisiopneumology Institute ‘Chilil Draganciuc’ in the Republic of Moldova and the National Centre for Disease Control and Public Health alongside the National Centre for TB and Lung Diseases in Georgia, operate under the Ministry of Health in their respective countries. They oversee the implementation of National TB programmes (NTP) through regular monitoring visits. In both countries, TPT is prescribed by TB specialists in private or public healthcare facilities. In the Republic of Moldova, HIV services coordinate TPT for PLHIV, whereas in Georgia, TPT for PLHIV is provided by TB network facilities.

### Study instrument

The data were collected using a semistructured interview guide, with questions grouped based on the case study’s conceptual framework—bureaucratic structure, communication, resources, disposition and role of individual actors. Following a review of various health policy analysis and implementation frameworks,^24^,^29^ Edward’s framework on policy implementation^28^ and Walt and Gilson’s health policy triangle^29^ were selected to be used in the case study for their proven utility in analysing health policy implementation.^30 31^ Edward’s framework provided the core conceptual foundation for analysing the policy implementation in TB prevention, with actor roles incorporated from Walt and Gilson’s policy triangle.

The guide was tested with a TB specialist from another country in the region and refined for better question flow.

### Data collection

The data were collected via Zoom videoconferencing during December 2023–May 2024. The respondents were purposefully selected to ensure diversity in organisational representation and roles, with the following inclusion criteria: service providers (TB, HIV, family doctors) and NTP staff; members of civil society organisations (CSOs) and employees of country-based international or donor organisations. They were identified by NTP managers, TB programme managers in the WHO Country Offices within participating countries and with the WHO Regional Office for Europe.

The respondents were invited by the lead author to participate in interviews through personalised email, wherein they received an invitation for interview with two language options: English or Russian. Following their initial agreement, participants were provided with the details of the study and asked to sign the consent form prior to interview. No incentives were provided for participation.

Data were collected, coded and analysed by the lead author, who was not involved in any activity related to TB prevention in these countries.

### Data coding and analysis

Transcripts generated based on recorded interviews were manually checked against the audio recording for accuracy, then edited, anonymised, deidentified and imported to NVivo V.14 for coding and analysis in the original language of the interview.

We applied a thematic analysis approach to analyse the data. Data coding occurred iteratively alongside data collection, with codes continuously refined and revised throughout the analysis. The codes within each theme, defined based on the study conceptual framework, were derived from the Inner Settings and Process domains of the Consolidated Framework for Implementation Research (CFIR), to guide our exploration of internal structures, within the health system, that shaped implementation processes, and served as the foundation for initial codes under each theme.^32^ Then, other codes pertaining to the theoretical model were added to the list. Additional codes and themes were added as they arose from the data.^33 34^ After coding, data within each theme were compared to analyse how the findings varied by implementation setting.

An important theme, patient attitudes towards TPT, emerged during the analysis, was incorporated in the study to deepen the insights of the findings.

### Patient and public involvement

Patients (not part of a stakeholder group) and the public were not involved in the design, conduct, analysis, reporting or dissemination of the results of this study.

## Results

### Participant characteristics

A total of 23 interviews were conducted: 11 in Georgia and 12 in the Republic of Moldova. Among the respondents, 14 were healthcare service providers or NTP personnel, 5 were from CSOs and 4 from the international or donor community. Many of the interviewees were female (table 1).

**Table 1:** Respondent characteristics

	Number of respondents		
	Republic of Moldova	Georgia	Total
Role			
TB services	4	3	7
HIV services	2	2	4
PHC services	1	2	3
CSOs	3	2	5
International/donor organisations	2	2	4
Gender			
Male	3	2	5
Female	9	9	18
Total	12	11	23

CSOs, civil society organisations; PHC, primary healthcare; TB, tuberculosis.

An average interview lasted 50 min. Seven interviews were conducted in English, 15 in Russian and 1 used a combination of both languages.

Two of the 26 identified potential respondents did not respond after the initial invitation and four reminders, and one interview was interrupted 10 min after the start and was not subsequently completed, due to non-response, so was discarded from the analysis. In two other cases of interruption, the connection was re-established, and interviews were successfully completed and included in the analysis.

The findings are presented based on the themes of the study’s conceptual framework, along with the theme that emerged through the interviews.

### Bureaucratic structures and monitoring mechanisms

In both settings, TB prevention services, including TPT, are incorporated into the broader health system. The Phthisiopneumology Institute ‘Chilil Draganciuc’ in the Republic of Moldova, and the National Centre for Disease Control and Public Health and the National Centre for TB and Lung Diseases in Georgia, oversee the implementation of the TB programmes, including TPT delivery. These institutional oversight structures maintain bureaucratic control through regular monitoring visits. Although these visits are intended to cover all aspects of TB care, their primary focus, as mentioned by almost all participants in both settings, is on contact identification, assessment of living conditions (with a focus on infection control) or TB treatment. Notably, none of the interviewees mentioned field monitoring visits addressing any aspects of TPT.

### Communication

Communication processes were similar across both study settings. No concerns or barriers were reported in formal and informal communication channels and information flow at all levels of healthcare providers in the two countries. All respondents perceived the communication process for implementing TB programmes as effective and unimpeded.

### Resources

#### Personnel

The personnel required for the implementation of TB prevention include TB, HIV and PHC service providers. In both settings, family doctors are responsible for screening the general population and people from high-risk groups for TB disease, referral for diagnostics and treatment.

TPT in both countries is prescribed by TB doctors, working in outpatient TB units, following laboratory testing and symptom screening or chest X-ray to rule out TB disease, except for PLHIV in the Republic of Moldova, for whom TPT is prescribed by HIV doctors. According to the majority of respondents, TB units are adequately staffed with well-trained personnel capable of providing a full range of TB services, including TPT. Participants in both settings believe that there is no shortage of TB doctors at any level.

However, a significant shortage of family doctors was reported in the Republic of Moldova. Despite this, many TB doctors suggest that the shortage of family physicians does not compromise the provision of TPT, as TPT is prescribed by TB doctors, while family doctors are in charge of its monitoring. However, the family doctor who participated in the study did not reflect on TPT monitoring as their responsibility.

According to TB doctors, in these situations, where there is a shortage of family physicians, the work is often partially redistributed to nurses, increasing their workload.

In most cases, I would say, not really [the shortage of family doctors compromises the provision of TPT] … [when family doctor is not available] the treatment is monitored by nurses, the TB outbreak spot is monitored by nurses. We just need doctors to check-in every 10 days, check the patients, to check their saturation, to examine them properly. TB doctor, Republic of Moldova

#### Availability of diagnostic equipment and tests

Healthcare providers in both countries reported using tuberculin skin test (TST) and interferon-gamma release assays (IGRAs) to confirm TBI. In Georgia, occasional lack of TST was reported by several interviewees, while in the Republic of Moldova, it was commonly available and widely used.

Sometimes we have [shortage of tests], for example, now we have the limitation in the TST. We have no Mantoux, but it’s not a problem [for TPT prescription]. TB doctor, Georgia

The IGRA test was only available at central levels in both settings; however, the majority of doctors do not consider it as a guidance or protocol-related hindrance to the rollout of TPT.

… it’s not obligatory to use the TST or IGRA tests. In the guideline we have such note that using of the IGRA or TST should not be defined as the barrier… If the country has no access to the IGRA or to the TST, even without testing, some of the risk groups should take the preventive therapy, if the active TB is excluded. TB doctor, Georgia

In both countries, the test is not mandatory for most of the high-risk groups, and logistical arrangements are in place to transport blood samples for testing to central facilities for those where need for additional testing and confirmation of TBI is identified for prescription of TPT.

#### Medication

Medicines, including those for new treatment regimens, are available, prioritised and used in both settings. No shortage of drugs was reported in either of the two countries.

### Provider disposition and attitudes

Almost all healthcare providers expressed positive attitudes towards TPT. According to the data collected in this study, the provider attitudes towards TPT evolved positively over time. The respondents noted that, in the past, they were less certain about prescribing the treatment, but their confidence has improved with experience and availability of short-course treatment regimens.

I would also say that when we started preventive therapy, that was in 2019, there was some fear from doctors to prescribe these drugs, because doses were very high, and they were very afraid of side-effects. But now we have some experience, and we are prescribing more like that. TB doctor, Georgia

However, it is noteworthy that some scepticism about TPT still persists among some TB doctors and family physicians. Several issues emerged, suggesting concerns about providers’ attitudes and confidence in prescribing the treatment. Several TB doctors, for example, mentioned that, while they are confident, some of their colleagues, especially family doctors, remain cautious about TPT, believing that the infection can resolve spontaneously in certain cases.

…they offered [TPT] to the patient but … it’s my feelings, that they think that human system will work themselves. TB doctor, Georgia

At the same time, TB specialists believe that the opinion of family doctors does not influence initiation or completion of TPT, as TB doctors are those who prescribe TPT.

They [family doctors] talk to me and say that they [family doctors] are not happy, but if I prescribe, they comply. TB doctor, Republic of Moldova

In both settings, providers exercise caution when prescribing TPT to children. In Georgia, TB doctors are hesitant about prescribing TPT without testing for TBI, although testing is not compulsory according to the national guidelines. In the Republic of Moldova, some TB doctors reported sending all children to Chisinau for additional consultation, testing and confirmation of the need for TPT.

In Georgia, where there is a shortage of TST, many providers also argue that confirming TBI with a test would enhance doctors’ confidence in prescribing TPT and patients’ adherence to it.

From the side of physicians, if the physician has diagnostic tests, like let’s say QuantiFERRON or Mantoux test, and they know the result is positive, they are sure and they are asking the patient to start preventive therapy. If they have no result they are in the trouble—what to do. It’s 50/50, to offer or not… TB doctor, Georgia

In Georgia, HIV care providers also noted that currently, according to the new guidelines and due to the introduction of new TPT regimens, they are temporarily not prescribing TPT to PLHIV. Instead, TB doctors at different health facilities manage TPT, creating an additional barrier for PLHIV, who must visit another facility for TBI treatment. According to the several interviewed providers, previously, when isoniazid preventive therapy (IPT) was used, HIV doctors confidently prescribed it themselves, which ensured continuity of care and made it easier for PLHIV to adhere to their treatment plans.

### Patients’ attitude

Outside of the health services, the primary factor significantly impacting the success of TPT scale-up is patients’ attitudes towards TPT. Though this question was not within the scope of this study, many providers in both settings reflected on patients’ attitudes and highlighted patients’ hesitancy to initiate TPT due to absence of TB symptoms, lack of confirmation of TBI or fear of side effects. Household contacts of current TB patients, for example, are reluctant to initiate the treatment that is similar to that prescribed to their family members with confirmed TB disease, especially if they are not experiencing symptoms or do not have test results confirming that they are also infected with TB.

… They [TB contacts] say “I should take this isoniazid and rifampicin and the person who is ill should take isoniazid and rifampicin too, why we should take the same treatment?” They have such questions… TB doctor, Georgia

### Role of individual actors

Interviewees in both settings described the implementation of TPT as being shaped by collaborative and systematic efforts of all individual actors involved in service delivery. Many participants emphasised that leaders, such as politicians, high-level officials or other public figures within or outside health services, played little or no active role on the rollout of TPT or other TB services.

## Discussion

This study revealed that challenges for the rollout of TPT across two settings are nearly identical. In both settings, there are established and well-functioning national structures, including adequately staffed TB units; effective communication mechanisms; and an uninterrupted supply of medicines for new treatment regimens – important enabling elements for TPT rollout. While both countries possess effective national-level infrastructures for oversight of TB programmes, the field monitoring visits predominantly emphasise TB diagnostics, treatment and contact identification, with less attention devoted to TPT, which indicates lower prioritisation of TB prevention, a problem that has been identified previously in other countries.^20 35 36^ Recognising the challenge, in 2024, the NTP in the Republic of Moldova, together with the National Medical Insurance Company, increased the number of field monitoring visits focusing on TB screening and TPT implementation. In addition to field monitoring visits, in both settings, TPT and TB screening data are monitored through existing information management systems.

In both settings, there is a need to strengthen the capacity of PHC services for TPT. In the Republic of Moldova, where TPT monitoring entails involvement of PHC, there might be discrepancies in the understanding of the roles and responsibilities for providing and monitoring TPT. While TB specialists believe that family doctors are responsible for TPT monitoring, this viewpoint was not cited by the interviewed family doctor. In Georgia, the responsibility for TPT is entirely within the TB system. As described by Matteelli *et al,*^37^ the lack or absence of systematic approaches to monitoring of TPT is common for many countries of the region.^37^ The PHC services in both countries face challenges such as staff shortages and high workloads. However, there is potential for increasing the role of family doctors in managing the full scale of TB preventive services. Through clarified roles, organised TB specialist support, adequate staffing and family doctors could better support TPT services.^38^ In both settings, this study was unable to comprehensively assess the PHC performance in TPT due to the limited participation of family doctors and the minimal information provided by them. This may be attributed to their low involvement in TB prevention relative to their high workload and broader range of responsibilities.

Another common challenge for TPT in both settings is the disposition and attitudes of healthcare providers towards TPT. Overall, the providers’ attitude to TPT is positive; however, TB and PHC doctors need increased confidence in prescribing TPT. Several other studies have identified provider attitude and confidence to TPT as a barrier for prescribing TPT.^3639^,^41^ Participants in our study suggest that confidence likely increases with provider experience, a finding also confirmed by HIV service providers in Georgia, who cited previous experience in confidently administering IPT. Additionally, enhanced support for clinical decision making (eg, test results) further strengthens providers’ confidence, as emphasised by healthcare providers in both countries.

With regard to testing for TBI before prescribing TPT, the approaches used in the two settings are different. WHO guidelines of management of TBI recommend that TST or IGRA tests can be used to test for TBI in all risk groups, except PLHIV or household contacts under 5 years old, for whom testing before initiation of TPT is not required.^9^ Our study identified that testing before initiation of TPT in Georgia is not required according to the national guidelines. It is done for certain groups (eg, children, patients with comorbidities) on an ad hoc basis. At the same time, the study showed issues with access to testing: TST was unavailable in Georgia at the time of study, and IGRA was only accessible at the central TB facility due to its high cost. While efforts are made to arrange delivery of samples for testing in some patients, this service is not provided to everyone. Although evidence is limited, other studies show that a test result with confirmed evidence of TBI can increase patients’ understanding of the disease risks and compliance to treatment,^42 43^ but more importantly, it may also enhance doctors’ confidence in prescribing TPT, as the findings from this study demonstrate for both settings.

### Study strengths and limitations

A primary strength of the study is that it was conducted in two settings with similar context but at different stages of implementation, highlighting similar challenges in TPT rollout at early and advanced stages of implementation of TB policies. This design enhances the transferability of findings and the potential to generalise the results, as well as to extend its conceptual framework and study methods across different contexts and environments.^44^,^46^ This is particularly important given a noticeable scarcity of the empirical studies on this topic specific to the European Region of WHO. Another key advantage of this case study is its methodological rigour, underscored by application of the methods of qualitative research, a tool of implementation research (CFIR), and a comprehensive, yet adapted theoretical framework, strengthening the reliability, credibility and applicability of findings. Additionally, in this study, we used an online format and bilingual approach to enhance inclusivity and reduce selection bias.

The study, however, has several limitations. First, the scope of this study did not allow for the evaluation of the challenges of TPT scale-up through patients’ viewpoints, an important challenge to TPT rollout, also shown in other settings.^13^,^15^ Second, healthcare providers’ self-reported attitudes on TPT may introduce bias, as responses could lean towards providing favourable or expected viewpoints. Additionally, the participation of PHC personnel in the study was limited, which restricted the insights to deeper understanding of their role in TPT provision. The study also did not focus on challenges of TPT scale-up among specific high-risk population groups, such as PLHIV, child and adult contacts of TB cases. Furthermore, the online format of the study may have limited the researcher’s ability to fully understand the context in which healthcare services are delivered. This is consistent with other scholars who find that observations and contextual factors that can be noted during in-person interactions could be somewhat missing in interviews conducted on distance.^47 48^ Lastly, neither the data collector nor the study participants were native speakers of the study languages, which may have affected the depth of some responses. Despite these limitations, this case study provides valuable insights into health system challenges of TPT rollout.

## Conclusions

Based on this case study, we conclude that the rollout of TPT—both at early and advanced stages of implementation—is supported by established system-based approaches for TB prevention and availability of TPT medicines that ensure that the treatment can be effectively delivered. While monitoring systems are in place, a greater emphasis on TB preventive measures would strengthen the efforts. Better integration of PHC and clearly defined and communicated role of family doctors in TPT is needed. Enhancing provider confidence in TPT remains a priority: although test results are not explicitly required for initiation of TPT under the existing protocols in both countries, their availability will support providers’ confidence to prescribe the treatment when needed and increase patients’ adherence to it.

### Recommendations

Based on the results of the case study, we recommend improving access to TBI testing to enhance providers’ confidence in prescribing TPT and support patient adherence. Strengthening oversight for TPT within existing monitoring systems is also essential to support TPT rollout. Additionally, optimising resource use and clarification of roles within PHC will ensure effective management of available personnel. Finally, expanding systematic TB screening in high-risk groups, with better integration of PHC and CSOs, will play a key role in successful rollout of TPT by increasing the number of people identified and their contacts referred to TPT.^23^

In addition to programmatic recommendations, we also suggest further research to explore in more depth the challenges associated with TPT delivery, including providers’ and patients’ attitudes across different high-risk groups and exploring risks to initiation of TPT due to negative TBI test results, potentially caused by suboptimal sensitivity of available tests.

## Data Availability

Data are available on reasonable request.
